# Acute Administration of Ojeok-san Ameliorates Pain-like Behaviors in Pre-Clinical Models of Inflammatory Bowel Diseases

**DOI:** 10.3390/nu15071559

**Published:** 2023-03-23

**Authors:** Emma A. Patton, Patrice Cunningham, Matthew Noneman, Henry P. Helms, Gustavo Martinez-Muniz, Aman S. Sumal, Milan K. Dhameja, Christian A. Unger, Ahmed K. Alahdami, Reilly T. Enos, Ioulia Chatzistamou, Kandy T. Velázquez

**Affiliations:** Department of Pathology, Microbiology and Immunology, University of South Carolina School of Medicine, Columbia, SC 29209, USA

**Keywords:** TNFα, mechanical sensitivity, aversion, conditioning place preference, colorectal distension

## Abstract

(1) Background: Gastrointestinal pain and fatigue are the most reported concerns of patients with inflammatory bowel disease (IBD). Commonly prescribed drugs focus on decreasing excessive inflammation. However, up to 20% of IBD patients in an “inactive” state experience abdominal pain. The medicinal herb Ojeok-san (OJS) has shown promise in the amelioration of visceral pain. However, no research on OJS has been conducted in preclinical models of IBD. The mechanism by which OJS promotes analgesia is still elusive, and it is unclear if OJS possesses addictive properties. (2) Aims: In this study, we examined the potential of OJS to promote analgesic effects and rewarding behavior. Additionally, we investigated if tumor necrosis factor alpha (TNFα) from macrophages is a primary culprit of IBD-induced nociception. (3) Methods: Multiple animal models of IBD were used to determine if OJS can reduce visceral nociception. TNFα-macrophage deficient mice were used to investigate the mechanism of action by which OJS reduces nociceptive behavior. Mechanical sensitivity and operant conditioning tests were used to determine the analgesic and rewarding effects of OJS. Body weight, colon length/weight, blood in stool, colonic inflammation, and complete blood count were assessed to determine disease progression. (4) Results: OJS reduced the evoked mechanical nociception in the dextran sulphate sodium model of colitis and IL-10 knockout (KO) mice and delayed aversion to colorectal distension in C57BL/6 mice. No rewarding behavior was observed in OJS-treated IL-10 KO and mdr1a KO mice. The analgesic effects of OJS are independent of macrophage TNFα levels and IBD progression. (5) Conclusions: OJS ameliorated elicited mechanical and visceral nociception without producing rewarding effects. The analgesic effects of OJS are not mediated by macrophage TNFα.

## 1. Introduction

An estimated 1.6 million U.S adults have been diagnosed with inflammatory bowel disease (IBD) [1,2]. Colitis is an IBD that is characterized by inflammation of the colorectum. Diarrhea, weight loss, and rectal bleeding are common side effects associated with extensive and long-lasting inflammation. A total of 70% of patients suffering from IBD will report abdominal and rectal pain [3,4,5]. This visceral pain (VP) will often dissipate as the patient recovers. However, approximately 20% of patients continue to experience this pain, even as the disease activity decreases [6]. Anti-Inflammatory treatments for IBD, such as corticosteroids, 5-aminosalicylic acid, biologics, and immunomodulators, often decrease the symptoms of the disease but have very little effect on the patient’s VP [7,8,9]. The use of common painkillers, such as narcotics and non-steroidal anti-inflammatory drugs, has been shown to aid in the management of VP, but has exhibited adverse reactions in the gastrointestinal system [10,11]. Furthermore, the use of narcotic painkillers presents a risk of inducing drug dependencies in patients [12,13]. Previous studies have found that an estimated 5% of all IBD patients may experience drug dependency [12,14]. Without a reliable and safe method to combat the VP associated with colitis, patients continue to suffer from the pain of the disease and the side effects of the treatment [15]. The utilization of novel therapies, such as complementary and integrative medicine, may elicit improvements in the treatment of VP in those with colitis.

Tumor necrosis factor alpha (TNFα) is a pro-inflammatory cytokine that can be produced by myeloid cells, such as macrophages (Mϕs) [16]. TNFα has been shown to play a role in the promotion of cancer pain, and exhibits upregulated expression in the later stages of colorectal cancer [17,18,19,20]. In patients with IBD, the increase of inflammation caused by TNFα can lead to a disruption in the integrity of the mucosal barrier, which is created by the interstitial epithelial cells of the colon [21]. Without this protective layer, the colon may experience excessive damage, emanating from a decreased ability to regulate homeostasis [22]. TNFα further prolongs the inflammation by activating the nuclear factor kappa B (NFκβ)-dependent pathways, which increase the expression of many cytokines and enzymes, and by upregulating other pro-inflammatory molecules, such as interleukin-1β and interleukin-6 [23]. Therefore, the association between Mϕs and TNFα production could be the key to understanding the pathogenesis of VP.

Ojeok-san (OJS), a medicinal herbal formula, is commonly used in Traditional Korean Medicine, Traditional Chinese Medicine (Wu-ji-san) and Kampo Japanese Medicine (Goshaku-san) as a way to combat pain, gastrointestinal problems, depression, and the common cold, among others [24,25]. Previous experimental studies that have utilized this remedy have shown to have anti-pyretic, anti-inflammatory, anti-metastatic, and analgesic properties [24,26,27,28,29,30,31,32]. These effects were observed with minimal to no discernable toxicity in the organs and cells of patients [27]. While the use of OJS may seem like the obvious choice for the treatment of VP in IBD, the mechanism by which OJS diminishes VP in patients is unknown. When used for in vitro studies, OJS has been shown to increase apoptosis and reduce inflammation through the activation of caspase-3 and a decrease in the expression of cytokines and macrophage-derived chemokines, respectively [31,33,34,35,36]. In fact, we have previously shown that OJS can reduce TNFα gene expression from macrophages in a dose-dependent matter [31]. Therefore, it is possible that OJS could ameliorate colitis and/or colitis symptoms, such as visceral hypersensitivity, by inhibiting TNFα release from macrophages.

Due to a number of studies that have shown a link between increased TNFα levels and pathogenesis of IBD [37,38,39], as well as the therapeutic potential of anti-TNFα therapy and soluble TNFα receptors [40], we sought to investigate the role of macrophage-secreted TNFα and OJS administration in colitis.

## 2. Material and Methods

### 2.1. Experimental Animals

Experiments conducted in this study were approved by the Institutional Animal Care and Use Committee (IACUC) of the University of South Carolina. The mice were held in ventilated cages and cared for in accordance with institutional guidelines (12 h:12 h light-dark cycle, 50% humidity, 22 °C temperature, low noise). All mice were a fed purified Ain76A diet ad libitum (Bio-Serv, Frenchtown, NJ, USA). Dextran sulfate sodium (DSS) was used to induce colitis in mutant mice that had TNFα deleted from their myeloid cells (TNFα ^flox/flox LysMcre^), as well as in littermate control WT mice. Ojeok-san was administered orally to investigate its role in mitigating colitis progression, histopathology, and visceral hyperalgesia. We used conditioning paradigms to determine as to whether OJS has rewarding effects in two models of spontaneous colitis (mdr1a constitutive knockout (multi-drug resistance genes, Abcb1a) and interleukin-10-deficient mice). Subsequently, we subjected C57BL6 (wild-type) mice to an aversive-conditioning test using colorectal balloon distension, in order to determine the non-reflexive analgesic properties of OJS.

#### 2.1.1. Experiment #1

Female and male TNFα ^flox/flox LysMcre^ and littermate control TNFα ^flox/flox^ mice were used in this experiment [41,42]. TNFα ^flox/flox^ mice have an increased production of TNFα (normal response), while TNFα ^flox/flox LysMcre^ mice exhibit a significantly blunted TNFα response to lipopolysaccharide, due to the decreased production of TNFα from myeloid cells, such as macrophages, monocytes, and granulocytes [41,42,43]. Mice (male and female, approximately 20 weeks old) were allocated into eight groups following stratified random sampling in terms of genotype, litter, sex, and body weight: TNFα flox/flox Control Vehicle, TNFα flox/flox DSS Vehicle, TNFα flox/flox Control Ojeok-san, TNFα flox/flox DSS Ojeok-san, TNFα flox/flox LysMcre Control Vehicle, TNFα flox/flox LysMcre DSS Vehicle, TNFα flox/flox LysMcre Control Ojeok-san, and TNFα flox/flox LysMcre DSS Ojeok-san. The experiments were conducted in four blocks. Water intake and disease activity index were assessed every day. Mechanical sensitivity to von Frey filaments was assessed on day 5. On day 7, mice were euthanized with an overdose of isoflurane, and their blood, plasma, and the colorectum was collected for further analysis.

#### 2.1.2. Experiment #2

Female and male mdr1a constitutive knockout mice were obtained from Taconic (Germantown, NY, USA). Mdr1a (multidrug resistance genes Abcb1a) KO mice have an intact immune system and develop spontaneous colitis, which is similar to human IBD, between 8 and 36 weeks of age [44,45]. By 20 weeks of age, all mdr1a KO mice developed colitis, which was determined by the presence of loose bloody stools (ColoScreen Tape, Helena Laboratories, Beumont, TX, USA). At 28 weeks of age, mdr1a KO mice were exposed to an automated Spatial Place Preference Box (Harvard Apparatus, Holliston, MA, USA), in order to determine the analgesic drug’s efficacy against spontaneous colitis [46]. Colon weight and length were measured, and blood was collected for cell blood count.

#### 2.1.3. Experiment #3

Interleukin-10 mutant (IL-10 KO) mice were originally obtained from Jackson Lab (B6.129P2-^Il10tm1Cgn/J^, Strain no. 002251) and bred at the University of South Carolina. This mouse genotype was selected due to the spontaneous development of chronic enterocolitis [47]. Regarding baseline mechanical sensitivity, young-adult (up to 12 weeks old) IL-10-deficient mice do not differ from WT mice [48,49,50,51]. Mechanical sensitivity was assessed at 7 months of age in male and female IL-10 KO mice. The mechanical threshold was defined as the force in grams, by which an animal reacts to 80% of the stimuli. Behavioral and metabolic phenotyping was conducted at approximately 8 months of age. The conditioned place preference test was performed at approximately 12 months of age. Body weight, colon weight, colon length, and blood were collected following euthanasia.

#### 2.1.4. Experiment #4

C56BL/6 mice were obtained from Jackson lab and bred at the University of South Carolina. Male and female C57BL/6 mice were used, due to their visceral sensitivity response to colorectal distension [52]. A conditioned place aversion test was performed at 24–25 months of age. Body weight was measured, and blood collected for complete blood count.

### 2.2. Dextran Sulphate Sodium (DSS) Colitis Development

For the chemical model of colitis, dextran sulfate sodium (DSS), was used due to the rapid development of colitis and visceral hypersensitivity [53]. DSS (2%) was obtained from TdB Labs (Uppala, Sweden), and supplied to the mice in their drinking water for a 5-day period.

### 2.3. Ojeok-san Administration

Ojeok-san (2 g/kg body weight) was obtained from Dr. Hyeun-Kyoo Shin (Director of the Herbal Medicine Formulation Research Group at the Korean Institute of Oriental Medicine). This herbal formula contains the following herbs: Atractylodis Rhizoma (*Atracylodes lancea D.C*., 7.5 g), Ephedrae Herba (*Ephedra sinica* Stapf, 3.75), Citri Unshius Pericarpium (*Citrus unshiu* Markovich, 3.75), Magnoliae Cortex (*Magnolia officinalis* Rehd. Et Wils., 3.0), Platycodonis Radix (*Platycodon grandiflorum* A. DC., 3.0) Aurantii Fructus Immaturus (*Citrus* unshiu Markovich, 3.0), Angelicae Gigantis Radix (*Angelica gigas* Nakai, 3.0), Zingiberis Rhizoma (*Zingiber officinale* Rosc., 3.0), Paeoniae Radix (*Paeonia lactiflora* Pall., 3.0), Poria Sclerotium (*Poria cocos* Wolf, 3.0), Angelicae Dahuricae Radix (*Angelica dahurica* Benth. Et Hook. F., 3.0), Cnidii Rhizoma (*Cnidium officinale* Makino, 2.63), Pinelliae Tuber (*Pinellia ternata* Breit., 2.63), Cinnamomi Cortex (*Cinnamomum cassia* Presi, 2.63), Glycyrrhizae Radix et Rhizoma (*Glycyrrhiza uralensis* Fisch. 2.25), Zingiberis Rhizoma Recens (*Zingiber officinale* Rosc., 3.75), and Allii Fistulosi Bulbus (*Allium fistulosum* L., 3.75). Mice were lightly anesthetized (mix of isoflurane and oxygen), then gavaged (22 g × 55 mm, Instech Laboratories, Plymouth Meeting, PA, USA) with 2 g/kg of Ojeok-san, either dissolved in water or vehicle (water). Mice were allowed to recover from the anesthesia for 10 min prior to any behavioral test [31].

### 2.4. Disease Activity Index, Histology, and Complete Blood Count

The disease activity index was calculated based on the sum of score-related symptoms (body weight loss (>5%WL = 0; 6–10%WL = 1; 11–15%WL = 2; 16–20% = 3, 21–25% = 4), stool consistency (pellet = 0; pasty = 2; liquid = 4), and blood in the stool (negative = 0, positive = 2, gross bleeding = 4)) [20]. The distal portion of the colorectum was dissected, weighed, measured, fixed in 4% paraformaldehyde, and stained with H&E and Alcian blue. H&E slides were evaluated (blinded to treatment groups) and scored according to the severity and extent of inflammatory cell infiltrate (1 = mild; 2 = moderate; 3 = marked), intestinal architecture, and epithelial changes (1 = focal erosions; 2 = erosions; 3 = extended erosions) by a certified pathologist (I.C.) [54]. Goblet cells were counted per six crypts and villus length was measured by Alcian blue-stained slides in a blinded fashion (P.C.). Blood was collected from the inferior vena cava using a heparinized needle and placed in a VetScan Hm5 (Abaxis, Union City, CA, USA) for hematology analysis. The blood was then centrifuged to separate the plasma, which was then stored at −80 °C for further testing.

### 2.5. Real-Time PCR

The proximal portion of the colon was used to determine the expression level of genes associated with pain (calcitonin receptor, calcr; mu opioid receptor, oprm; kappa opioid receptor, oprk) and colitis (interleukin-6, IL-6; nucleotide-binding oligomerization domain-containing 2, NOD2; TNFα; interleukin-4 (IL-4)) using qRT-PCR. Briefly, colonic RNA isolation was performed using trizol, chloroform, and isopropanol, and samples were purified with lithium chloride [55]. The RNA concentration and purity were assessed using a NanoDrop (ThermoFisher, Waltham, MA, USA). An iScript cDNA Synthesis Kit (Bio-Rad, Hercules, CA, USA) was used to convert RNA into cDNA. Bio-rad probes were used to determine the expression of pain and colitis markers, using TATA box binding protein (TBP), ubiquitin C (UBC), and glyceraldehyde-3-phosphate dehydrogenase (GAPDH) as housekeeping genes.

### 2.6. Behavioral Tests

The mechanical sensitivity to von Frey filaments (0.008, 0.02, 0.04, 0.07, 0.16, 0.40, 0.6, 1.0, 1.4, 2, 4, 6 g) was assessed on day 5 (10 min after oral administration of Ojeok-san or water), after a 3 day habituation period [31]. The mechanical threshold was defined as the force in grams at which any von Frey filament elicited 5 nociceptive responses (jumping, sharp retraction from the abdomen, and immediate licking or scratching of the lower abdomen) out of 5 stimuli. All mechanical sensitivity tests were performed between 7–9 am (beginning of light cycle) by the same investigator, who was blinded to the treatment groups. Additionally, the experimenter analyzing the data was blinded to group assignments.

The conditioned place preference (CPP) test was conducted using an automated Spatial Place Preference Box (Harvard Apparatus, Holliston, MA, USA), in order to examine the affective component of pain in colitis. This paradigm was conducted over a total of 4–5 weeks. Prior to the beginning of the first trial, an initial place preference of each mouse was obtained by allowing each mouse free range within the three-compartment Spatial Place Preference Box for 15 min. Chamber preference (baseline) was assessed by an automated weight transducer technology, which detected the animal‘s position. For experiment #2, two conditioning trials (45 min each/day for two days) were performed in the morning (mice conditioned to the preferred side, with oral administration of water) and in the afternoon (mice conditioned to non-preferred side, with oral administration of 2 g/kg body weight of Ojeok-san or water). During the test days, (drug-free) mice were allowed to move freely through the three-compartment chamber (15 min). Four or five drug free preference tests were conducted after 4 days of conditioning trials. Preference testing tests were conducted between 7:30 a.m. and 9:30 a.m. Conditioning trials were performed between 7 a.m. and 11 a.m. for morning conditioning and between 11 a.m. and 3 p.m. for afternoon conditioning. Four consecutive conditioning trials and preference tests were conducted. The percentage of time spent in the non-preferred (Ojeok-san conditioning) compartment was calculated as follows: time (s) spent in the non-preferred compartment, divided by the time (s) spent in the three compartments (preferred, non-preferred, and corridor), multiplied by 100. The total number of entries into the compartments was also calculated, in order to assess general locomotor activity. In experiment #3, we modified the conditioned place preference protocol due to the number of animals and time constraints. After assessing chamber preference, animals were conditioned (oral gavage of water and/or Ojeok-san) for four days to the non-preferred chamber for 45 min. On the 5th day (drug-free preference testing) of each conditioned trial, mice were allowed free range to the Special Place Preference Box and the time spent in each chamber was monitored.

Behavioral and metabolic phenotyping were assessed for nine days using the Promethion System (Sable Systems International, Las Vegas, NV, USA). Prior to placing the mice in the automated Promethion System, a single dose of oral Ojeok-san (2 g/kg of body weight) and/or vehicle (water) was administered to IL-10 KO mice. Ambulatory locomotion time, still time, and sleep time were obtained, in order to determine the effects of OJS on spontaneous behaviors during the light and night cycles.

A conditioned place-aversion experiment (automated Spatial Place Preference Box from Harvard Apparatus, Holliston, MA, USA) was performed to examine if OJS can hinder the aversive features of colorectal distension (Barostat, Distender Series II, G&J Electronics) [56]. During baseline measurements, mice were allowed to explore the three-compartment Spatial Place Preference Box for 15 min. Four conditioning trials of approximately 60 min/day were performed prior to each test day (Tests 1–5). The repeated phasic distension protocol consisted of twelve 55 mmHg pressure stimuli, with a 20 s duration each and with 5 min rest (non-inflated balloon) intervals. Mice were anesthetized (isoflurane) prior to balloon insertion and the administration of Ojeok-san (2 g/kg of body weight) and/or water via oral gavage. Mice were allowed to recover from the anesthesia for 10 min prior to the start of conditioning with an aversive stimulus (colorectal balloon distension). Drug-free/CRD-free testing was conducted for 15 min, during which mice were allowed to move freely through the three-compartment chamber. The percentage of time spent in the preferred chamber was graphed. Preconditioning (baseline), conditioning trials, and drug-free tests were conducted between 7 a.m. and 11 a.m. The percentage of time spent in the preferred (aversion conditioning) compartment was calculated as follows: time (s) spent in the preferred compartment divided by the time (s) spent in the three compartments (preferred, non-preferred, and corridor), and multiplied by 100.

### 2.7. Data Analysis

Data were analyzed using GraphPad software, Prism 8.0. A three-way ANOVA (disease × genotype × treatment) with a Fisher’s LSD post hoc analysis was used to assess body weight, colon length, colon weight, histopathology, villus length, goblet cell count, gene expression, immune cell count, and mechanical threshold outcomes in Experiment #1. Water intake and the disease activity index in Experiment #1 were analyzed using a mixed-effects model followed by Fisher’s LSD. For Experiments #2, 3, and 4, colon weight, colon length, and complete blood count were analyzed using unpaired Student’s *t*-tests. The conditioned place-preference and conditioned place-aversion tests were analyzed using a two-way RM ANOVA, followed by Fisher’s LSD. Statistical data that did not pass the equal variance test (Barlett’s Test) were logarithmically transformed and further analyzed. The level of significance was set at *p* < 0.05 and the data are presented as means standard error.

## 3. Results

### 3.1. Macrophage TNFα Deletion and Ojeok-san Administration Do Not Impact Disease Progression in a Chemical Model of Acute Colitis

**Figure 1:**
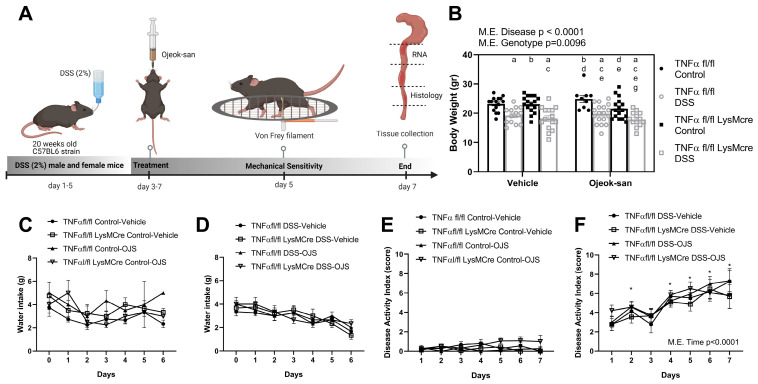
Macrophage deletion of TNFα and Ojeok-san administration does not impact disease progression in a chemical model of acute colitis. (**A**) Graphical depiction of the experimental design, (**B**) body weight, (**C**) water intake in control animals, (**D**) water intake in DSS-treated mice, (**E**) disease activity index in control animals, and (**F**) disease activity index in DSS-treated mice. Values are represented as mean ± SEM; *n* = 10–18 mice per group. Three-way ANOVA for B. Letters indicate statistical significance (*p* < 0.05) from ^a^ TNFα fl/fl Control-Vehicle, ^b^ TNFα fl/fl DSS-Vehicle, ^c^ TNFα fl/fl LysMcre Control-Vehicle, ^d^ TNFα fl/fl LysMcre DSS-Vehicle, ^e^ TNFα fl/fl Control-OJS, ^f^ TNFα fl/fl DSS-OJS, ^g^ TNFα fl/fl LysMcre Control-OJS. Mixed-effects model followed by an uncorrected Fisher’s LSD post hoc test for C–F. * Indicates statistical difference from the TNFα fl/fl DSS-Vehicle group on day 1.

To determine the impact that OJS administration and macrophage TNFα deletion (TNFαfl/fl-LysMcre) have on disease progression, we assessed body weight, disease activity index, colon length and weight, histopathological score, villus length, and goblet cell number (Figure 1A). As expected, mice that received DSS exhibited a significant decrease in body weight when compared to TNFα fl/fl Control-Vehicle (WT) (Figure 1B, *p* < 0.05). There was a main effect of the TNFαfl/fl-LysMcre genotype, regarding a decrease in body weight. Regarding OJS administration, no main effect of treatment was observed. No changes in water intake were observed between treatment groups (Figure 1C,D). No changes in the disease activity index were observed in mice that did not receive DSS (Figure 1E). A main difference in time was found for the disease activity index for all treatment groups that received DSS (Figure 1F, *p* < 0.05), indicative of a typical colitis progression (body weight loss, diarrhea, and blood in stool).

**Figure 2:**
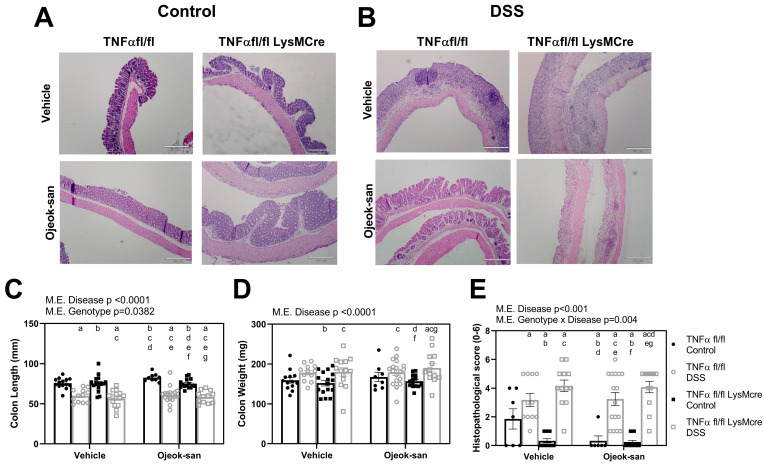
Ojeok-san administration does not impact colon size and histopathology in a chemical model of acute colitis. (**A**) Representative H&E staining of colonic tissue from control and (**B**) DSS-treated mice, (**C**) colon length, (**D**) colon weight, and (**E**) colonic histopathological score following acute DSS administration and OJS treatment in TNFα fl/fl and TNFα fl/fl-LysMCre mice. Values are represented as mean ± SEM; *n* = 8–18 mice per group. Three-way ANOVA followed by uncorrected Fisher’s LSD post hoc test. Letters indicate statistical significance (*p* < 0.05) from ^a^ TNFα fl/fl Control-Vehicle, ^b^ TNFα fl/fl DSS-Vehicle, ^c^ TNFα fl/fl LysMcre Control-Vehicle, ^d^ TNFα fl/fl LysMcre DSS-Vehicle, ^e^ TNFα fl/fl Control-OJS, ^f^ TNFα fl/fl DSS-OJS, ^g^ TNFα fl/fl LysMcre Control-OJS.

Figure 2A,B are representative colon tissue H&E images from control and colitis mice, respectively. There was a main effect of the disease (colitis), in terms of decreasing colon length, increasing colon weight, and exacerbating inflammation in mice with colitis (Figure 2C,E, *p* < 0.0001). TNFαfl/fl-LysMcre Control-OJS mice displayed a shorter colon length relative to TNFαfl/fl Control-OJS mice (*p* = 0.0382). An interaction between genotype and disease was found to decrease the inflammatory score in TNFαfl/fl-LysMcre Control-Vehicle mice when compared to TNFαfl/fl Control-Vehicle mice (*p* = 0.004).

**Figure 3:**
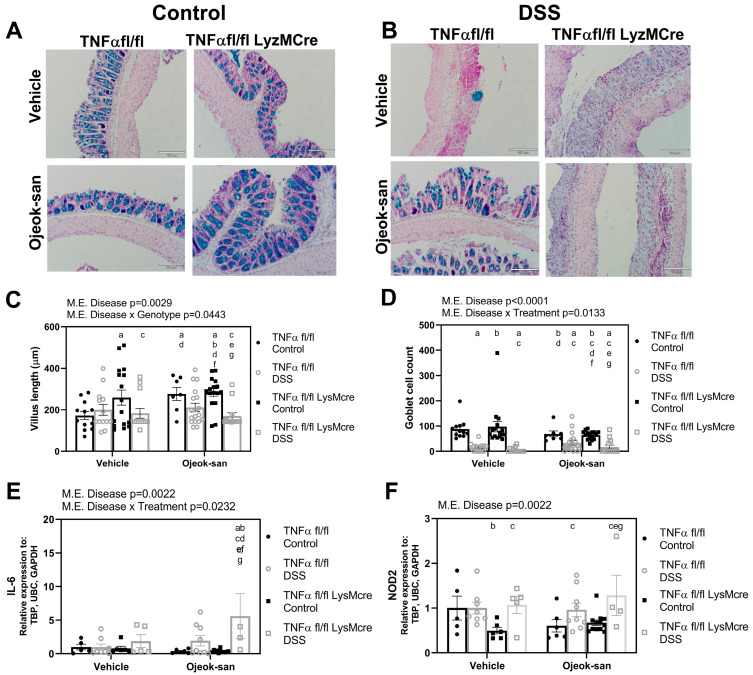
Macrophage deletion of TNFα influences villus length, and Ojeok-san administration influences goblet cell number and colonic IL6 expression. (**A**) Representative Alcian blue staining of colonic tissue from control and (**B**) DSS-treated mice; (**C**) Villus length, (**D**) goblet cell count, (**E**) IL-6 gene expression, and (**F**) NOD2 gene expression following acute DSS administration and OJS treatment in TNFα fl/fl and TNFα fl/fl-LysMCre mice. Values are represented as mean ± SEM; *n* = 6–16 mice per group. Three-way ANOVA followed by an uncorrected Fisher’s LSD post hoc test. Letters indicate statistical significance (*p* < 0.05) from ^a^ TNFα fl/fl Control-Vehicle, ^b^ TNFα fl/fl DSS-Vehicle, ^c^ TNFα fl/fl LysMcre Control-Vehicle, ^d^ TNFα fl/fl LysMcre DSS-Vehicle, ^e^ TNFα fl/fl Control-OJS, ^f^ TNFα fl/fl DSS-OJS, ^g^ TNFα fl/fl LysMcre Control-OJS.

Goblet cells in the colon tissue were visualized using an Alcian blue stain (Figure 3A,B). The average number of goblet cells was determined per six crypts, and villus length was measured. DSS treatment was found to decrease villus length and goblet cell number in colitis mice (Figure 3C,D, *p* < 0.001). An interaction between disease and genotype was found to result in an increased villus length in TNFαfl/fl-LysMcre Control- Vehicle and OJS-treated mice, as well as TNFαfl/fl Control-OJS mice, relative to TNFαfl/fl Control-Vehicle mice, suggesting that TNFα deletion from macrophages has an impact on villus length (*p* = 0.0443). An interaction between disease and treatment was found to decrease goblet cells in Control-OJS-treated TNFαfl/fl-LysMcre mice relative to Control-Vehicle-treated TNFαfl/fl-LysMcre mice (*p* = 0.0133). However, no difference was detected in the number of goblet cells between TNFαfl/fl Control-OJS and TNFαfl/fl DSS-OJS mice. DSS treatment was found to increase the gene expression of inflammatory genes, interleukin-6 (IL-6) and nucleotide-binding oligomerization domain-containing 2 (NOD2) (Figure 3E,F, *p* = 0.022), but no significant changes were observed in terms of TNFα and interleukin-4 (IL-4) levels in the colon (Supplementary Figure S1). An interaction between disease and treatment was found to increase IL-6 expression in TNFαfl/fl-LysMcre DSS-OJS mice relative to all groups (*p* = 0.0232).

### 3.2. Macrophage Deletion of TNFα Does Not Impact the Blood Profile but Ojeok-san Administration Diminishes Lymphocyte Levels in Mice with Colitis

**Figure 4:**
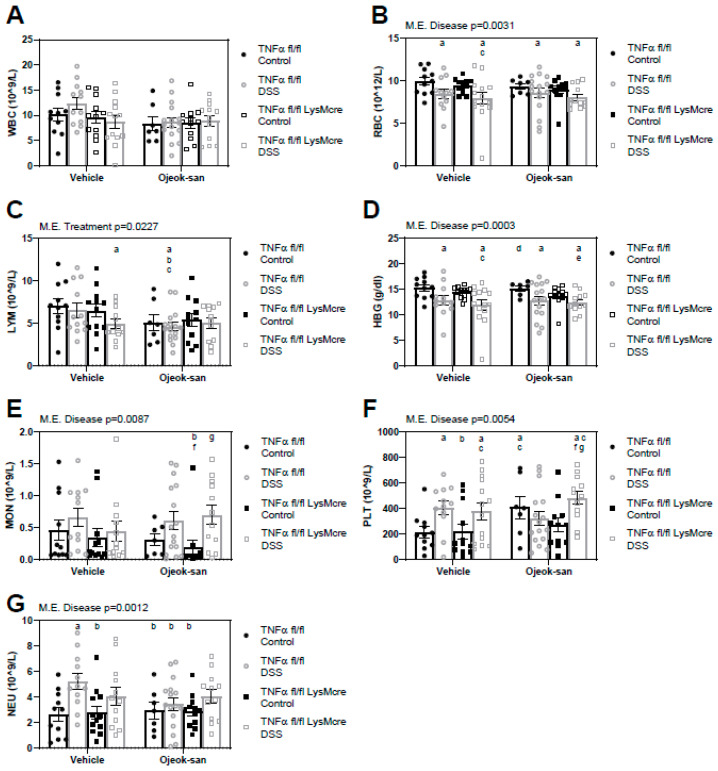
Macrophage deletion of TNFα does not impact blood profile, but Ojeok-san administration diminishes lymphocyte levels in mice with colitis. (**A**) WBC, (**B**) RBC, (**C**) LYM, (**D**) HGB, (**E**) MON, (**F**) PLT, (**G**) NEU following acute DSS administration and OJS treatment in TNFα fl/fl and TNFα fl/fl-LysMCre mice. Values are represented as mean ± SEM; *n* = 7–18 mice per group. Three-way ANOVA followed by uncorrected Fisher’s LSD post hoc test. Letters indicate statistical significance (*p* < 0.05) from ^a^ TNFα fl/fl Control-Vehicle, ^b^ TNFα fl/fl DSS-Vehicle, ^c^ TNFα fl/fl LysMcre Control-Vehicle, ^d^ TNFα fl/fl LysMcre DSS-Vehicle, ^e^ TNFα fl/fl Control-OJS, ^f^ TNFα fl/fl DSS-OJS, ^g^ TNFα fl/fl LysMcre Control-OJS.

To examine the impact that OJS administration and macrophage TNFα deletion has on the blood profile, a complete blood count was performed (Figure 4A–F). A consequence of the disease was an increase in monocytes (MON, *p* = 0.0087), neutrophils (NEU, *p* = 0.0012), and platelets (PLT, *p* = 0.0054), and a decrease in red blood cells (RBC, *p* = 0.0031) and hemoglobin (HBG, *p* = 0.0003). A main effect of treatment was a decrease in the number of lymphocytes (*p* = 0.0227) in the blood of TNFαfl/fl DSS-OJS mice relative to TNFαfl/fl Control-vehicle, TNFαfl/fl DSS-Vehicle, and TNFαfl/fl-LysMcre Control-Vehicle mice. Furthermore, lymphocyte levels were decreased (LYM, *p* = 0.0227) in TNFαfl/fl-LysMcre DSS-Vehicle mice when compared to TNFαfl/fl Control-Vehicle mice.

### 3.3. Deletion of Macrophage TNFα Does Not Impact Mechanical Sensitivity but Ojeok-san Administration Improves Mechanical Hyperalgesia in Mice with Colitis

**Figure 5:**
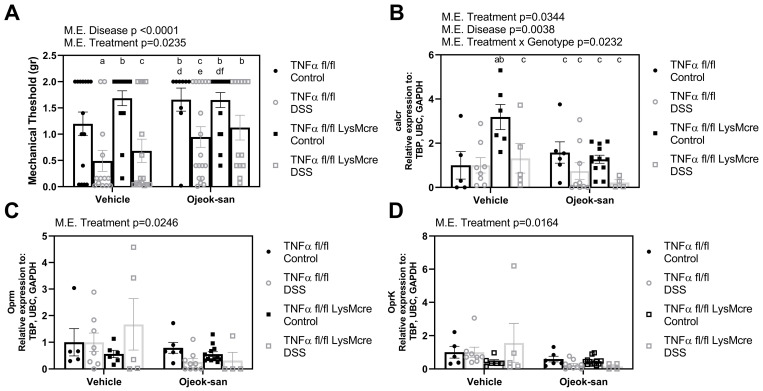
Macrophage deletion of TNFα does not impact mechanical sensitivity, but Ojeok-san administration improves mechanical hyperalgesia in mice with colitis. (**A**) Mechanical threshold to stimuli with von Frey filaments; gene expression of (**B**) calcr, (**C**) oprm, and (**D**) oprk following acute DSS administration and OJS treatment in TNFα fl/fl and TNFα fl/fl-LysMCre mice. Values are represented as mean ± SEM; *n* = 4–18 mice per group. Three-way ANOVA followed by uncorrected Fisher’s LSD post hoc test. Letters indicate statistical significance (*p* < 0.05) from ^a^ TNFα fl/fl Control-Vehicle, ^b^ TNFα fl/fl DSS-Vehicle, ^c^ TNFα fl/fl LysMcre Control-Vehicle, ^d^ TNFα fl/fl LysMcre DSS-Vehicle, ^e^ TNFα fl/fl Control-OJS, ^f^ TNFα fl/fl DSS-OJS, ^g^ TNFα fl/fl LysMcre Control-OJS.

To assess the mechanical sensitivity and gauge the response of the mice to nociception, mechanical threshold to von Frey filaments was measured. Effects of both disease and treatment were detected in the mice during the nociceptive behavior sensitivity test (Figure 5A, *p* < 0.05). Mice with colitis exhibited increased mechanical sensitivity (lower mechanical threshold) and DSS mice treated with OJS showed decreased mechanical hyperalgesia (increased mechanical threshold). We next addressed whether OJS is capable of controlling pain signaling at the level of the nociceptors. Both the disease and treatment were found to decrease calcitonin receptor (calcr) gene expression (Figure 5B, *p* < 0.05). Interestingly, an interaction between the treatment and genotype (*p* = 0.0232) was found to increase calcr gene expression in the colon tissue of TNFαfl/fl-LysMcre Control-Vehicle mice. Gene expression of the μ-opioid receptor (oprm) and κ-opioid receptor (oprk) was also examined (Figure 5C,D). OJS treatment was found to decrease the gene expression of oprm and oprk (*p* < 0.05).

### 3.4. Four Weeks of Ojeok-san Administration Does Not Impact Disease Progression nor Show Rewarding Properties in the mdr1a KO Mouse Model of Spontaneous Colitis

**Figure 6:**
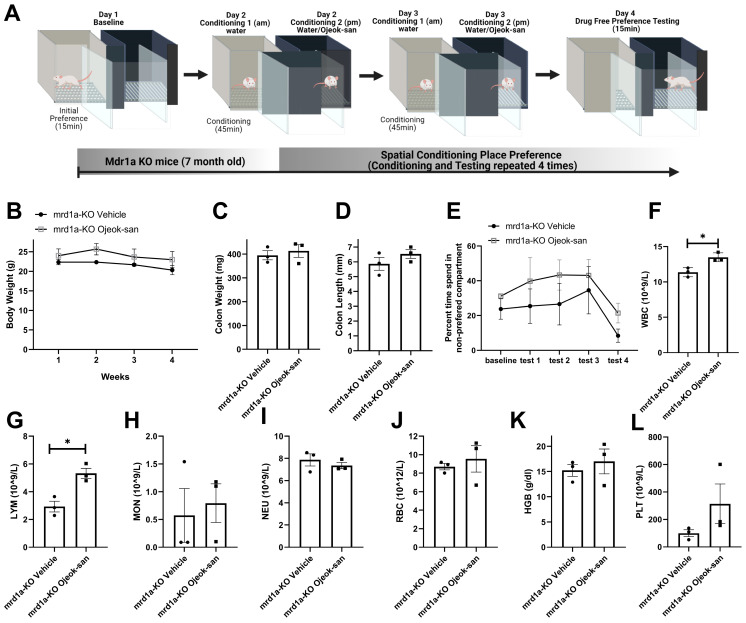
Four-week administration of OJS does not impact disease progression or show rewarding properties in the mdr1a KO mouse model of spontaneous colitis. (**A**) Graphical depiction of spatial conditioning place-preference protocol; (**B**) body weight, (**C**) colon length, (**D**) colon weight, (**E**) percent time spend on the OJS-paired side compartment during the test sessions, (**F**) WBC, (**G**) LYM, (**H**) MON, (**I**) NEU, (**J**) RBC, (**K**) HGB, and (**L**) PLT in vehicle- and OJS-treated mrd1a KO mice. Values are represented as mean ± SEM; *n* = 3 mice per group. Two-way RM AVOVA was conducted for (**A**,**E**). Two-tail unpaired *t*-test was conducted for (**C**,**D**), and (**F**–**L**). * Indicates statistical significance between groups, *p* < 0.05.

To evaluate whether OJS has the potential to become addictive, we utilized a spatial conditioned place-preference paradigm to assess OJS’s rewarding properties. For this test, we first determined initial preference for one compartment versus the other, then OJS administration was paired with the non-preferred compartment (Figure 6A). Rewarding properties are associated with changes in compartment preference. Regarding disease progression, a four-week administration of OJS did not change body weight, colon weight, or colon length in mrd1a-KO mice (Figure 6B,C). No difference was detected in the percentage of time spent in the non-preferred compartment among mrd1a-KO vehicle and mrd1a-KO OJS treated mice (Figure 6E). To examine if a longer OJS exposure has an impact on the blood profile, we performed a complete blood count. An increase in WBC and LYM was observed in mrd1a KO OJS-treated mice when compared to mrd1a KO vehicle mice. No differences were detected between groups with respect to MON, NEU, RBC, HGB, and PLT counts.

### 3.5. Five Weeks of Ojeok-san Supplementation Does Not Impact Disease Progression but Improves Mechanical Hyperalgesia without Promoting Rewarding Behavior in an IL-10 KO Mouse Model of Spontaneous Colitis

**Figure 7:**
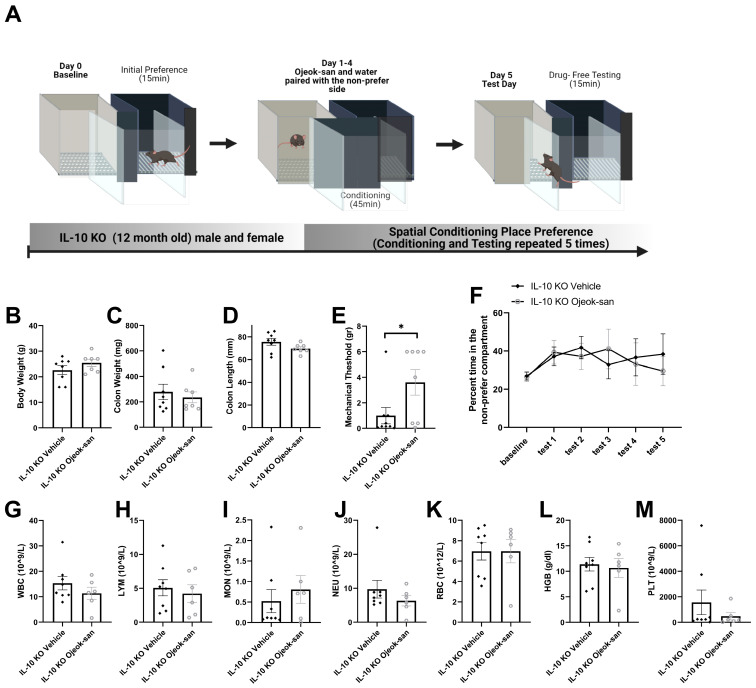
Five-week administration of OJS does not impact disease progression, but improves mechanical hyperalgesia, without promoting reward-seeking behavior in the IL-10 KO mouse model of spontaneous colitis. (**A**) A graphical depiction of spatial conditioning place-preference protocol, (**B**) body weight, (**C**) colon length, (**D**) colon weight, (**E**) mechanical threshold to stimuli with von Frey filaments, (**F**) percentage of time spent on the OJS-paired side compartment during the test sessions, (**G**) WBC, (**H**) LYM, (**I**) MON, (**J**) NEU, (**K**) RBC, (**L**) HGB, and (**M**) PLT in vehicle- and OJS-treated IL-10 KO mice. Values are represented as mean ± SEM; *n* = 8–9 mice per group. Two-way RM AVOVA was conducted for F. Two-tail unpaired *t*-test was conducted for (**B**–**E**), and (**G**–**M**). * Indicates statistical significance between groups, *p* < 0.05.

To confirm the impact of OJS on colitis, analgesia, and rewarding behavior, we utilized the IL-10 KO mouse model of spontaneous colitis (Figure 7A). No differences were observed in body weight, colon weight, or colon length between the IL-10 KO vehicle and IL-10 KO OJS mice (Figure 7B–D). IL-10 KO OJS-treated mice showed an elevated mechanical threshold when compared to IL-10 KO vehicle mice (Figure 7E). Regarding the addictive potential of the analgesic, there was no reward-seeking behavior observed with OJS treatment in IL-10 KO mice (Figure 7F). No differences were detected between IL-10 KO vehicle and IL-10 KO OJS mice with respect to WBC, LYM, MON, NEU, RBC, HGB, and PLT.

**Figure 8:**
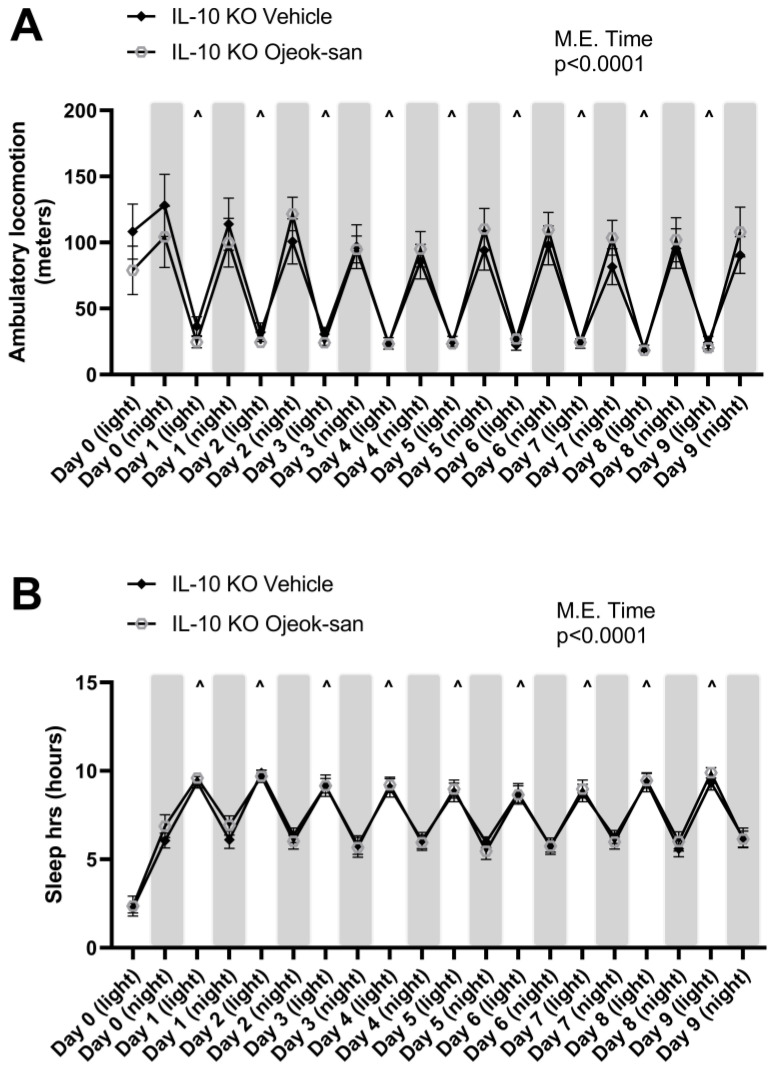
A single dose of OJS does not impact locomotor activity or sleep behavior in an IL-10 KO mouse model of spontaneous colitis. (**A**) Distance that IL-10 KO vehicle- and OJS-treated mice travelled in their home cage during the light and night cycle, (**B**) Time that the IL-10 KO vehicle- and OJS-treated mice spent sleeping during the light and night cycle. Values are represented as mean ± SEM; *n* = 8 mice per group. Two-way RM AVOVA. ^ *p* < 0.05 Indicates statistical significance between night and light cycle.

We have previously reported that OJS exhibits sedative properties in mice with colorectal cancer [31]. However, we do not know for how long this effect can last. Therefore, we orally administered OJS (as a one-time gavage on day 0) or water (vehicle) into IL-10 KO mice to examine locomotive, still, and sleeping behaviors. As expected in nocturnal animals, an increase in locomotive activity during the night and sleep activity during the day was found over time (Figure 8A,B). However, no differences were detected in locomotion and sleep time between IL-10 KO vehicle and IL-10 KO OJS mice after a single administration of OJS.

### 3.6. Ojeok-san Reduces Aversive Behavior Produced by the Colorectal Balloon Distension in C57BL6 Mice

**Figure 9:**
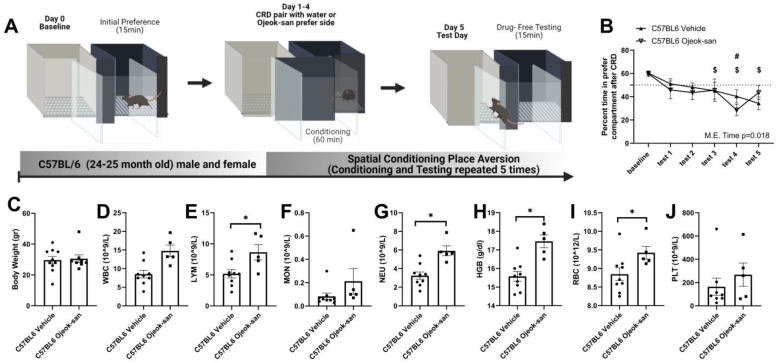
OJS reduces aversive behavior produced by colorectal balloon distension in C57BL6 mice. (**A**) Graphical depiction of spatial conditioning place-aversion protocol, (**B**) percentage of time spend in the preferred (non-OJS-paired side) compartment during the test sessions, (**C**) body weight, (**D**) WBC, (**E**) LYM, (**F**) MON, (**G**) NEU, (**H**) RBC, (**I**) HGB, and (**J**) PLT in vehicle- and OJS-treated C57BL6 mice. Values are represented as mean ± SEM; *n* = 7–10 mice per group. Two-way RM AVOVA for B. ^$^ Indicates statistical significance from baseline in C57BL6 Vehicle mice. ^#^ Indicates statistical significance from baseline in C57BL6 OJS-treated mice. Two-tail unpaired *t*-test for (**C**–**J**), * *p* < 0.05.

Colorectal distension is the gold standard method for evaluating colon sensitivity in animals and humans [57,58,59]. A positive correlation between pressure and visceromotor response (abdominal musculature contraction) has been associated with increased pain perception in humans [56,57,59,60]. We have previously shown that OJS reduces visceral nociception in a pre-clinical model of colorectal cancer [31]. However, non-reflexive (operant) measurements have not been used to determine the analgesic properties of OJS. We tested OJS using a conditioned place-aversion paradigm, since colorectal distension has been shown to produce aversive behavior when using a passive avoidance behavioral paradigm [56]. Colorectal distension at a pressure of 55 mmHg elicited avoidance behavior (i.e., mice spend less time in the preferred compartment during the test day) in three out of five tests (Tests 3, 4 and 5) in C57BL6 mice that were administered water (Figure 9A,B, *p* < 0.05). However, mice that received OJS instead of vehicle (water) during conditioning training only showed avoidance behavior in one of the five tests (Test 4) (*p* < 0.05). No differences were detected in terms of body weight, WBC, MON, or PLT between C57BL6 Vehicle and C57BL6 OJS mice (Figure 9C–D,F,J). A five week administration of OJS in C57BL6 mice increased LYM, NEU, HGB, and RBC levels in the blood (Figure 9E,G–I).

## 4. Discussion

Gastrointestinal pain and fatigue are the most reported concerns of patients with IBD [5,61]. Commonly prescribed drugs for IBD focus on decreasing excess inflammation without effectively managing patients’ pain [7,8,9]. However, approximately 20 percent of the IBD patients in an “inactive/remission” state experience abdominal pain [6,62]. Acute and/or chronic visceral pain in patients with IBD continues to be undertreated, and the prescription of narcotics after hospitalization continues, despite opioid use being associated with increased inpatient stays, infection, and death [15,63,64]. Therefore, alternative pain relievers should be explored, given that patients continue to feel the negative effects of the disease and the current treatments. Since we have previously reported that OJS ameliorates visceral and somatic nociception [31], and preclinical models of IBD have already been established to promote visceral nociception in rodents [65,66,67], we proposed to investigate if OJS can serve as a non-addictive alternative treatment to reduce IBD-associated nociception. We found that OJS reduces mechanical and visceral IBD-associated nociception, without causing rewarding effects. Although TNFα has been identified to be an important mediator of IBD and pain development [68,69,70], mice with macrophage-derived TNFα deletion did not exhibit any decreased disease severity, including that of nociceptive behavior.

Ojeok-san has been shown to decrease macrophage-mediated inflammatory processes and reduce the production of TNFα [33,34]. Its anti-pyretic, anti-inflammatory, anti-metastatic, and analgesic properties make OJS a good candidate for the future treatment of IBD-associated pain [24,26,27,28,29,30]. The data gathered in this study show that OJS is able to decrease mechanical hyperalgesia in a chemically induced and a genetic model of a spontaneous colitis. Additionally, OJS reduced visceral, non-reflexive nociception using a conditioned place-aversion paradigm. Nociceptive behaviors have been previously observed to occur at the peak of colitis in both DSS-treated and IL-10 KO mice [67,71]. However, nociception sensitivity is not present at late and early time points in these models [48,49,50,51,72]. Some possible explanations might be due to the endogenous production of opioids by T cells in IL10-decificent mice [73] and the lack of colitis in young IL-10 KO mice. Previously, we have shown that OJS can reduce both somatic, referred hyperalgesia, as well as visceral sensitivity to a colorectal distension of 40–80 mmHg [31]. The analgesic effects of OJS appeared to be indirect, as OJS was not able to reduce dorsal root ganglia firing in vitro [31]. We and others have shown that OJS reduces TNFα gene expression in macrophages, as well as tissue and plasma TNFα levels [31,32]. Therefore, we hypothesized that OJS decreases nociception by diminishing TNFα production.

Based on our findings, we conclude that the anti-nociceptive effects of OJS are not mediated via TNFα originating from macrophages. In fact, macrophage-derived TNFα does not contribute to somatic referred hyperalgesia, and might play a small role in colitis development and progression. Nonetheless, others have found that TNFα is an important mediator in the pathogenesis of ulcerative colitis, and it is also involved in increased neuronal excitability in dorsal root ganglia innervating the colon [23,39,74,75]. For instance, it is possible that TNFα is secreted from other cells rather than macrophages, as T and B lymphocytes, epithelial, and endothelial cells can produce TNFα [76,77,78,79,80]. More importantly, treatments using anti-TNFα have shown to be effective early on but less effective in inducing remission in ulcerative colitis cases [81,82,83,84]. Similar to our results, De Santis et. al. showed that whole-body TNFα deficiency does not influence colitis development in the Winnie mouse model of ulcerative colitis [85]. Furthermore, utilizing DSS in a whole-body TNFα deletion mouse, Naito et. al., suggested that the blockage of TNFα can be detrimental, as it can lead to a decrease in survival and enhance intestinal inflammation [86]. Evidence of a compensatory increase of interleukin-1β in TNFα-deficient Winnie mice might explain why anti-TNFα therapy decreases efficacy [85]. As such it is possible that the antinociceptive effects of OJS might be caused by diminishing other IBD-associated nociceptive signals, such as transient receptor potential vanilloid 1 (TRPV1), substance P, and the calcitonin gene-related peptide (CGRP), to mention a few [87].

When administered Ojeok-san, mice displayed a more tolerant response to mechanical stimulation with von Frey filaments, as well as less aversive behavior to colorectal distension. These data suggest that the presence of OJS in the body decreases the nociceptive behaviors associated with colitis and visceral pain. Based on the data gathered, OJS administration decreases the colonic expression of calcitonin receptors, μ-opioid receptors, and κ-opioid receptors. The calcitonin receptor is the functional receptor of CGRP, which is known to mediate colonic motor responses, pain, and inflammation [88,89,90]. Previous studies have shown that deletion or blockage of the calcitonin receptor in rats with IBD (trinitrobenzene sulfonic acid, TNBS) exacerbated colonic inflammation and necrosis [91,92]. Whether the impact of OJS on calcitonin receptor expression is influencing inflammation, muscle tone, and/or nociception is not known. Regarding opioid receptors in colitis, an increased expression of μ-opioid receptors has been associated with a protective role against inflammation [93,94]. However, another study found no changes in μ-, δ-, and κ-opioid receptors in a model of colitis [95]. In our study, we did not observe an increase in histopathological inflammation or nociception in OJS-treated mice. Therefore, it is unclear as to how OJS is affecting opioid receptors.

Given that drug dependence and opioid misuse has been observed in patients with IBD [13,96,97], we screened OJS for potential abuse liability using conditioned place-preference tests. We found no differences in compartment preference between vehicle and OJS-treated mdr1a KO and IL-10 KO mice. Addictive substances such as morphine, cocaine and amphetamine have shown rewarding properties (preference for paired compartment) using the conditioned place-preference test [98,99,100]. In recent attempts to determine analgesic efficacy of drugs in animal studies, conditioned place-preference paradigms have been used, as pain relief is associated with a rewarding experience [46]. Testing analgesic efficacy with the conditioned place preference assay might bring unintended consequence, as addictive substances are extremely rewarding. Additionally, rats treated with the painkiller indomethacin failed to produce place preference [46]. Nonetheless, the use of multiple paradigms that test nociception via eliciting stimuli, spontaneous painful diseases, rewarding behavior, and aversive states might be necessary to advance pain management.

Overall, the data we gathered indicate that OJS has potential as an analgesic compound to ameliorate visceral sensitivity. However, the direct mechanism by which OJS decreases nociception associated with colitis is still unknown. Further research will be needed to determine if the effects of OJS are mediated via the peripheral or central nervous system. By continuing to research OJS, we may be able to further understand how this herbal formula works and properly apply it as a safe and reliable treatment to ameliorate pain.

## Data Availability

Data regarding this work are available from the corresponding author upon request.

## Supplementary Materials

The following supporting information can be downloaded at: https://www.mdpi.com/article/10.3390/nu15071559/s1, Figure S1: Macrophage deletion of TNFα and Ojeok-san administration do not influence colonic TNFα and IL-4 expression.
