# Supplementary material for: Acute Administration of Ojeok-san Ameliorates Pain-like Behaviors in Pre-Clinical Models of Inflammatory Bowel Diseases
Source: Nutrients. 2023 Mar 23;15(7):1559. doi: 10.3390/nu15071559 (PMC10096710; doi:10.3390/nu15071559)
Supplement: Supplementary file 1 [file nutrients-15-01559-s001.zip › nutrients-2237733-supplementary.pdf]

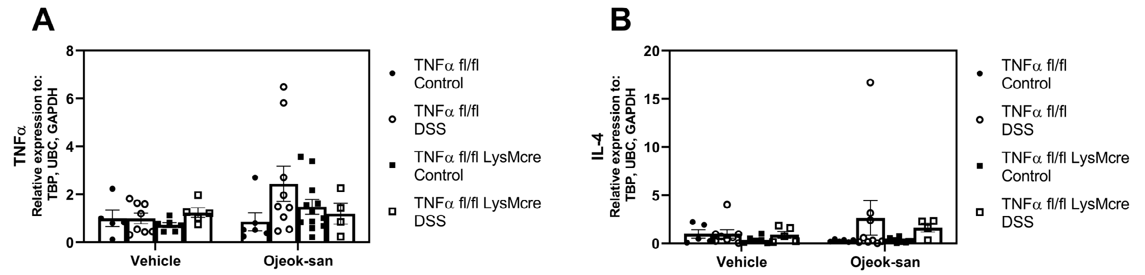

Figure S1. Macrophage deletion of TNFα and Ojeok-san administration do not influence colonic TNFα and IL-4 expression.
